# Institutional community engagement leader perspectives on supporting ethical community-engaged research

**DOI:** 10.1017/cts.2024.1165

**Published:** 2025-01-06

**Authors:** Stephanie Solomon Cargill, Nancy Shore, Rachel Olech, Phoebe Friesen, Jessica Rowe, Sana Khoury-Shakour, Emily E. Anderson

**Affiliations:** 1 Saint Louis University, St Louis, MO, USA; 2 University of New England, Portland, ME, USA; 3 University of Illinois Chicago, Chicago, IL, USA; 4 McGill University, Montreal, QC, Canada; 5 Yale University, New Haven, CT, USA; 6 University of California, Santa Cruz, CA, USA; 7 Loyola University Chicago, Chicago, IL, USA

**Keywords:** Community-engaged research, Institutional Review Board, Clinical and Translational Science Award, community engagement, interviews, qualitative

## Abstract

**Introduction:**

Over the last couple of decades, there has been a growing awareness of the value of community-engaged research (CEnR). Simultaneously, many academic institutions have established centralized support for CEnR. For example, dozens of academic medical centers in the United States receive National Institutes of Health (NIH)-funded Clinical and Translational Science Awards (CTSAs) and have embedded community engagement programs (CE) whose primary expertise and mission is to advance CEnR at their institutions.

**Methods:**

As part of a larger interview study aiming to learn more about how institutional CE programs and HRPPs work together, we analyzed interviews with CE program leaders at academic medical centers that receive funding from the NIH CTSA program to identify barriers and strategies to conducting CEnR at their institutions, primarily focusing on the relationships with Institutional Review Boards (IRBs).

**Results:**

We identified three categories in the interviews: barriers and strategies vis-à-vis IRBs to address 1) CE/IRB relationships; 2) Understanding issues; and 3) Structural and resource issues.

**Conclusions:**

CTSA CE program leaders have experience implementing solutions to common barriers to IRB review faced by CEnR researchers. The barriers they face in these three categories and the strategies they use to overcome them can provide helpful insights to others who hope to facilitate CEnR research at their institutions.

## Introduction

Over the last couple of decades, there has been a growing awareness of the importance of centering community voice in shaping health research and facilitating its translation into practice. Community-engaged research (CEnR), an umbrella term that we define broadly as the active involvement of communities in research that affects their interests, is becoming increasingly frequent and familiar [1].

More research funders are requiring community engagement, including the National Institutes of Health (NIH), the Patient Centered Outcomes Research Institute, and the Bill and Melinda Gates Foundation. Research institutions are making changes to be more responsive to the needs of CEnR. IRBs are becoming more familiar with CEnR approaches and more knowledgeable about how to apply inherent flexibilities in the federal regulations to CEnR, such as recognizing the qualifications of community partners to conduct research and adapting prospective reviews for emergent designs [2,3]. Importantly, consensus is emerging about the ethical principles guiding CEnR, such as close collaboration, trust, mutuality, shared power, decision-making, and data ownership, as well as the obligations of researchers who conduct this type of research [4].

Additionally, many academic institutions have established centralized support for community engagement (CE) more broadly, which includes CEnR as well as other community engagement functions (e.g., university-community “relations” or “partnerships” for governance, funding mechanisms, educational partnerships, etc.). Dozens of academic medical centers in the United States (U.S.) receive NIH-funded Clinical and Translational Science Awards (CTSAs), constituting a national network of medical institutions that aim to accelerate the translation of research discoveries into improved healthcare and health [5]. Community engagement is a required component for CTSA funding [6]. These CTSA CE programs aim to “accelerate clinical research, expand treatment delivery and speed the response to public health challenges [7].” Although methods of and resources dedicated to CE vary across CTSA-funded institutions, CE is a central component.

Despite this expansion of support for CEnR, scholars have argued that there are aspects of the traditional research enterprise that continue to be a poor fit for CEnR [8,9]. Successful CEnR requires adequate resources, which may not always be available from funders, and sufficient time, which may be at odds with the promotion and tenure clock at academic institutions [10]. The ideal timeline of CEnR is also often at odds with how research is conventionally funded and reviewed; while in CEnR, engagement should ideally begin before the parameters of the project have been settled, in reality, resources are usually not available until a specific project has been funded. Silos at academic institutions or policies that have not kept up with the changing research environment, such as those that impact academic researchers’ ability to fairly compensate community research partners, can impede the type of flexibility demanded by CEnR [11]. Some researchers who attempt CEnR may not be adequately trained [12,13]. Central to this discussion, CEnR advocates have frequently discussed how Human Research Protection Programs (HRPPs) and institutional review boards (IRBs) can pose barriers to CEnR research; for example, by questioning the role or qualifications of community partners or hindering its iterative approach by requiring prospective review [3,14–18].

CTSA CE program leaders have experience implementing solutions to common barriers faced by CEnR researchers. As part of a larger study aiming to learn more about how institutional CE programs and HRPPs work together (as well as how they might collaborate more), we interviewed CE program leaders at academic medical centers that receive funding from the NIH CTSA program. The primary aim of the study was to develop consensus recommendations for engaging community perspectives in the research review and human research protections (publication under review). In this manuscript, we focus on strategies of CE program leaders and their staff for overcoming barriers to appropriate IRB review of CEnR at their institutions. These insights generated from a relatively well-supported context should be uniquely valuable in indicating where additional resources should be focused to address persistent barriers.

## Materials and methods

### Participants and recruitment

In Fall 2021, we sent an email invitation to leader(s) of CE programs at all 62 lead institutions funded by NIH CTSA awards inviting them (or their designee) to participate in an in-depth interview on connections between the HRPP and institutional CE functions/activities. Email addresses for these individuals were publicly available on institutional websites. Individuals interested in participating could respond by clicking a link that directed them to a RedCap survey where they could select a day/time for their interview. The study was approved by the IRB at the University of Illinois Chicago and received a waiver of written informed consent.

### In-depth interview and procedures

The interview guide was developed by the authors, who all have experience and expertise in ethical issues in CEnR and/or human research protections. While the primary purpose of the study was to examine how IRBs include participant and community perspectives in their review, we also asked CE program leaders to share their perspectives on the unique ethical challenges that arise for community-engaged research(ers) – including but not limited to IRB review – and how their institutional CE program supports investigators to address these challenges. Table 1 includes sample interview guide questions.

**Table 1.** Sample community engagement leader interview guide questions

Please describe how you and your CE program or team interacts [or has interacted] [formally or informally] with your IRB/HRPP. What is your sense of the relationship between researchers doing patient-engaged or community-engaged research and the IRB? On what issues related to human research protections and/or IRB review do you advise investigators? What resources are available? Can you think of a specific time when researchers came to you because they encountered the IRB as a barrier to CEnR? How if at all did you support these investigators? How could the IRB better support researchers doing patient-engaged or community-engaged research? What additional resources are needed? Do you have any other thoughts on how you see your CE program/team’s role in promoting ethical research?

CE = Community Engagement; CEnR = Community Engaged Research; IRB = Institutional Review Board; HRPP: Human Research Protections Program.

Interviews were conducted over Zoom by one of the authors (RO) and were recorded and auto-transcribed. After each interview, a research assistant reviewed the recording and the transcript to ensure accuracy and redact identifying information (e.g., institution name) prior to final storage for analysis. Participants were offered the opportunity to review their transcript, although all declined this offer. Interviews were conducted until we reached saturation of themes [19]. A total of 17 interviews were conducted. Interviews averaged 46 minutes and ranged between 33 and 66 minutes.

### Analysis

We conducted a thematic analysis of the transcripts [20]. Two authors (SSC, NS) reviewed the data in order to identify “barriers,” defined as factors interviewees identified as impeding the conduct of CEnR, and “strategies,” defined as factors interviewees identified as supporting and promoting CEnR. After reading an initial set of transcripts, the two coders met and agreed upon an initial set of themes identified as strategies and barriers, and then divided the remaining transcripts to independently code. Coding discrepancies were then reconciled through discussion during consensus meetings. While coding the transcripts for strategy and barrier themes, each coder started to identify possible groupings among the themes. After extensive discussion and engagement with the existing literature, the authors divided the themes into three primary codes, with both barriers and strategies falling under each code. Due to the nature of the overall interviews, the final codebook reflected only barriers and strategies between CEnR and the HRPPs/IRBs, although interviewees sometimes mentioned other institutional barriers and strategies outside the HRPP/IRB.

## Results

The resulting codebook reflected three primary codes, each of which encompassed both barrier and strategy themes. This was not surprising as many interviewees discussed strategies emerging as a result of experiencing particular barriers. The first was coded as “CE/IRB relationship” and referred to the dynamics between those committed to CE and those who worked at the IRB. The second was coded as “Understanding issues” and were themes reflecting the need for IRBs to truly understand CEnR in order to appropriately review it. The third category of codes was called “Structural and resource issues” and captured themes that emerged from the institutional structure of IRB review of CEnR. Each code will be discussed with corresponding quotes below.

### CE/IRB relationship

Many interviewees identified the nature of the relationship between IRBs and CEnR stakeholders ( researchers, community representatives, etc.) as a primary barrier to conducting CEnR at their institution. Specifically, several interviewees perceived that their IRBs have negative attitudes toward CEnR. This included a perception that CEnR is less rigorous than other research approaches:

*…it’s sort of adversarial because I feel like every time we’re doing a study and putting it forward we’re also doing education about here’s what our science is, here’s why our science is this way - it is different from this other thing. And so, I just feel like my protocols end up being a lot longer than others, not because you need that content, but because you’re educating the reviewers about what that science looks like.*

Interviewees also commented on negative IRB perspectives on community input/involvement:

*[T]here’s a lot of disrespect of community perspectives, and I think if we were to do this [consider community perspectives] in a meaningful way and people would have the experiences that I’ve had from doing community engaged work, like how much I’ve learned from the community, how many perspectives and ideas and insights I’ve gotten that I would never have come to on my own, even if I sat here reading my journals all day. I think that that [disrespect] would dissipate, but I think there would be a lot of resistance [to such a change]*

Inversely, interviewees also discussed “negativity toward everything about the process of IRBs” coming from those doing CEnR.

*…everyone’s complaining about how much time [IRB review] takes and all that kind of stuff and I’m like, but the thing is you’re not the expert here - you might think you are, but you’re not. The IRB analysts and IRB itself know the regulations in and out…*

*[T]here’s so much of disdain for having to deal with their IRB sometimes, because it’s gonna take forever. So, there’s all this negativity, negativity toward everything about the process of IRBs.*

Related to this bidirectional tension, interviewees discussed problematic communication between IRBs and CEnR researchers during the submission and review process, disparagingly described by one interviewee as a “digital relationship” with communication entirely through online systems and email. Others described feedback from the IRB as excessive or appearing to come out of nowhere, a common complaint from researchers regardless of methods, discipline, approach, or institution.

Strategies proposed by interviewees to improve the relationship between IRBs and CEnR stakeholders focus on promoting early and engaged direct (not solely digital) communication and collaboration that manifested patience, transparency, and flexibility.

*I feel like over the years, even prior to our [CTSA award], with some of the things that we did with community engagement - there was no protocol, there was no template, there was no anything. So, I was the one who is calling them and saying, “All right, this is what I’m trying to do, I don’t know how to set it up within this format,” and they would work with me to figure out how to do that. I think they’re incredibly flexible and incredibly…. they want to be able to figure this stuff out.*

*I actually met folks in the IRB and have felt comfortable having conversations and of voicing concerns. And if the application has been submitted, instead of calling whomever my reviewer is, I can ask someone else that I have that relationship with for the assistance and getting it done. And so, I don’t think that researchers at our institution have that relationship, which is probably impossible for everyone to have, but to have a contact at the IRB who certainly focuses on developing those relationships with researchers, I think it would be beneficial.*

Beyond building relationships through direct and personal communication about one’s studies, some interviewees found it effective to bring CEnR researchers and IRB staff together.

*…we just invited the director of the IRB to be involved in one of our meetings that takes place on a monthly basis, and the goal of that meeting is to improve under-invited participants in research, to make the people that say yes to research, and that are involved in each project, be truly representative of the disease that is being researched*

[The IRB] has invited me to their standing meeting…

Finally, several people mentioned the benefits of having a “champion” for CEnR within the IRB, for example:

*…[someone] that has been a little bit more involved with the type of studies, community engaged studies. So then that way, we’re able to explain it and then they’re like, “Oh yes, I get that now.” And as they’re seeing more of it, it makes more sense and it makes it a little bit easier [of a] process.*

### Understanding issues

Beyond improving relational connections between IRBs and CEnR stakeholders, the second category of barriers and strategies brought up by interviewees was around IRB understanding related to supporting and reviewing CEnR. Interviewees noted that IRBs often fail to appreciate that the ethical values underlying CEnR expand upon and fulfill the ethical obligations articulated in the Belmont Report, particularly the often-overlooked obligations towards justice.

*The individual people in an IRB program don’t always understand or appreciate the extent to which research would be made better with community perspectives. And I think that changing that orientation would be really useful… and that means training, which means resources - that means changing the expectation of the IRB program like it’s not just about balancing risk and benefit, but about addressing justice in a proactive way … You can’t just make sure that studies aren’t harmful, you have to also make sure that studies are helpful, right - that they advance justice or that they advance health.*

Interviewees also noted that IRBs may lack expertise to review CEnR protocols, given unique methodological considerations and approaches. For example, IRB reviewers may not understand the roles of community partners on study teams and the value of their contributions, the importance of collaboration with community organizations, and the need for community-friendly resources and support.

*…(O)ur IRB …know(s) how to review a clinical trial. When you get into community relationships and community based participatory research in areas that are more implementation science, there’s not as much expertise in terms of the reviewing pool for it, and so we end up with a lot of questions that are just things that, frankly, if you had experts that were doing it, they would know those are not the things.*

*Deciphering what is the partnership, versus what is the research, and what is the data collection, because sometimes those things get muddied depending on the reviewer. They’re not sure like “your community advisory board, you’re recruiting?,” “no we’re not recruiting those people, those are partners, those are people who are going to be advising the study” “but then you’re recruiting the participants?” I think in our heads when we do that type of work it’s very clear, whose role is what, but depending on the experience and the expertise of the IRB reviewer, they may or may not know the nuances and it just gets jumbled into one pot.*

As institutional advocates for CEnR, interviewees discussed their strategies to increase their IRBs’ understanding of CEnR. Many mentioned designing or implementing trainings on CEnR specifically for IRB staff and members. Another strategy was inviting IRB staff and members to attend or co-present at training programs on CEnR offered to the broader research community. Finally, some interviewees provided case-by-case consultations with IRB reviewers.

*We’ve done a lot of work to educate the IRB on what community-engaged research is, how it’s different allowing the IRB to understand that they need to have a little bit of latitude to allow for some of the iterative nature of community-engaged work.*

*We’ve been meeting regularly with IRB staff around some very some specific issues around covering community sites that don’t have their own IRB and kind of defining types of projects and the ways that will work or won’t work.*

It is worth noting that although many of the interviewees noted positive experiences educating IRBs about CEnR, several mentioned that this should not be the responsibility of CEnR researchers and CE administrators.

*… it shouldn’t be our role because our role should be to link folks directly with the community, and that our role is as a liaison with the community and not as a training entity. Ideally, I think that IRB would do a good job of that in-house. I don’t think they do a job of it at all, and therefore we will probably fill in that gap as community engagement often does end up filling in the gap. But I don’t think it should be our responsibility - we don’t have the resources for it, we don’t have the staffing or the funding or anything like that for it.*

Interviewees also noted barriers arising from IRBs not understanding the burden that traditional human research protections trainings pose to community research partners. The content of such trainings were considered by several interviewees to be irrelevant or inaccessible to communities, yet it was also noted that some IRBs do not have the necessary background or skills to provide more appropriate training for community research partners. Many of the interviewees were familiar with the flexibility IRBs have to accept alternative trainings, as well as the existence of trainings designed specifically for CEnR contexts. A primary strategy used to offset this barrier was to educate IRBs on the alternatives to existing trainings and their regulatory flexibility in accepting them. Some interviewees framed this as a matter of justice:

*The impetus behind that was I was working with community partners, for whom access to the CITI (Collaborative Institutional Training Initiative) modules, both because of the digital divide, but also because the literacy level is so text heavy, that I was like I can’t do this. And it was in the context of some work with tribal communities and asking our tribal partners to complete the CITI modules, right, and they’re like … the layers of historical oppression, there you know, yeah like it was really like this is not going to fly.*

### Structural and resource issues

Many of our interviewees recognized that even with positive relationships and adequate understanding to review CEnR, IRBs are functioning with limited bandwidth, and appropriately reviewing CEnR often places additional time and resource burdens where they are already lacking.

*What I hear from the IRB is, it’s a lot about bandwidth - so there’s a lot they would like to do, but I think that they feel like they can just kind of barely keep their head above water, as it is, with all that’s required of them.*

One barrier frequently mentioned under this category was the burden of having to “reinvent the wheel” each time CEnR is brought to the IRB. While it is expected that the first time an IRB/HRPP encounters a novel issue, resolution will take time, experience should inform similar challenges and decisions in the future. However, interviewees frequently described experiences in which a CEnR protocol was reviewed on a case-by-case or ad-hoc basis, without being informed by the similar CEnR questions that have arisen and been addressed previously.

Specifically, interviewees identified the barriers presented by lack of institutional memory and personnel turnover.

*It continues to be a hurdle because there’s been so much turnover at our IRB … In the last, you know just in the midst of this pandemic, I’ve bumped into reviewers [who] have been like, “what are you talking about - you can’t add a non [university] person to a protocol.” And I was like “yes, I can and here’s like this decade of history to show that I can… And so, there is this loss of institutional memory that has actually made it harder to add community members as investigators.*

*[Community engagement] can be very dependent on the commitment of the [IRB] leadership and so changing leadership will mean going back to the beginning because none of this is really institutionalized yet.*

Another barrier for CEnR researchers mentioned by interviewees was the lack of a mandate and thus no regulatory guidance and limited incentive to incorporate CEnR considerations into IRB review.

*[IRBs] don’t have any mandate to look at how solid a community engagement plan is or … to consider the rights of a community and how those might be addressed. So, if that was a mandate sure, I think then, educating them would be helpful.*

Many interviewees indicated strategies that addressed these structural issues. Some mentioned working with HRPPs to implement structural changes to incentivize appropriate and efficient review of CEnR protocols. Several interviewees reported that their IRB had formalized the process to obtain community input on research and included this input in their IRB submission. Models included a formal IRB referral mechanism to CE programs on their website or directly from the IRB or even creating a standing community advisory board made up of lay (non-scientist) individuals representing the local communities from which research participants are recruited that the IRB could consult regarding individual protocols as well as policies and forms.

*“The IRB does have a community engagement group or community engagement committee that they have formed to be thinking about these issues, and I am now sort of a liaison between our community engagement core and the regulatory team that’s thinking about these issues. So, they have been really wonderful over the last year, very inclusive, inviting me to be part of their conversations.”*

*“The community group, more of what I would call a much more grassroots community-oriented group, that would work directly with the IRB staff … around website about language, communications, promotions, information there.”*

Another group of structural changes reported or recommended by interviewees was to integrate CEnR considerations into the submission and review process to IRBs, rather than engaging them alongside them or separately.

*“Asking folks who are submitting applications to have a brief section that describes their community engagement plan if they’re doing a community-based study. … I think we feel like that will at least get researchers thinking about if they’re doing community-based work, if there is an element of community engagement - how do you build that into the protocols that are being submitted.”*

*“I’ve heard about other CTSAs who have documents or requirements by their IRB to include a community engagement plan for dissemination for researchers … It’s for everybody… it might be hard to push, it might be hard for someone to just to grasp on to but it’s a move in the right direction.”*

It was also suggested that the referrals could be embedded in the IRB submission process.

*“A couple things that we’ve talked about that are on our list would be like specific language in [IRB] outcome letters and in the application that would guide researchers to us at an early phase to community engagement … like hey, there’s a support infrastructure to engage community, please go to them and consult with them and come back when you have. . .*

A final structural change that reflects the discussion of trainings for community partners above was not only having IRBs *accept* alternative trainings, but officially and explicitly endorsing alternative trainings.

*“I just vetted the CIRTification program* [alternative training in human research protections training tailored for community research partners] *with both children’s IRB and [university name] IRB and got official sign-off … we now formally present this as the accepted training for community members. And you know, kind of certified training, for when community has, or when researchers have community members on their staff.”*

In addition to structural changes, many interviewees discussed resources that they developed to make CEnR review more streamlined. Some CE programs were in discussions with IRBs or had actually developed with IRBs, templates, and guidance documents. One CE program developed a flow diagram to articulate the types of projects and partnerships that would be covered under their current IRB structure. A second CE program developed a guidance document:

*We worked with one of the directors that we spoke with and had meetings with, and also our Community Advisory Board to come up with a guidance document around considerations that researchers and also for IRB to consider when creating a proposal for community engaged research. It consisted of different questions - some around recruitment, some around funding, things like that.*

Another resource mentioned by an interviewee was called a “landscape analysis” of particular geographical areas or communities that are solicited for research participation.

*[W]e worked with community partners and leaders to develop an assessment system for really gauging the feasibility of incoming research. What we’ve developed is kind of a landscape -it’s just a two page summary about those regions that talks about cancer and politics, and economics, and environment, and technological, and social, you know - resources or gaps. And that’s just to give to the researchers, like if you really want to go put a research study in this area, you need to understand this about the region. . .. And then it’s kind of like red light/yellow light/green light, “hey this looks really exciting to our region, but investigator, can you do this this and this?” and, if not, it’s probably not going to be successful, so do you really want to spend your time kind of mucking around over here if you’re not going to get what you need out of enrollment and recruitment, and community buy-in and all that kind of stuff?*

Although intended to benefit researchers, this would also be a relevant resource for IRBs by incorporating community considerations relevant to the proposed research.

Interviewees also discussed institutional barriers to CEnR beyond the IRB. Some noted that IRBs might leverage their power to serve as advocates for broader institutional change related to CEnR, for example around the issue of compensating partners that are often shaped at the institutional level.

*It’s extremely difficult to pay [community partners] and sometimes it’s state regulations, sometimes it’s university, your academic regulations. But it’s hard to pay them, and it’s important that we do - they have an expertise. Researchers expect to get paid; the community member sharing their expertise should expect to get paid, and if the IRB could do something with that, with their powerful pull, that would be advantageous.*

## Discussion

Previous research identified tensions and challenges between IRBs and CEnR researchers [2,3,14,15]. With the proliferation of CEnR as a requirement for funding and integration of CE across the clinical and translational research spectrum, centralized/institutional CE programs have also grown. These CE programs, unlike many other institutions, have resources and personnel devoted to facilitating CE throughout the university. The findings of this project, which explored the views of these personnel, yield two important points. First, it is noteworthy that even well-resourced and experienced CE program leaders continue to face barriers to IRB review at multiple levels. This suggests that such barriers are likely entrenched throughout research institutions given that they are still experienced even among the most supported CE programs in the country. This may be because federal funders’ requirements for CE in research do not yet have the weight to spur institutional change because CEnR is still not that common, or simply because large academic research institutions are resistant to change in general.

Second, CE programs have been hard at work trying to address these barriers in ways that other institutions with less support and experience with CE may not yet have had the opportunity. Data from this study indicate that CE programs are in the process of enacting strategies to forge healthy relationships between IRBs and CEnR researchers, building CEnR competencies within their institutions, and initiating structural changes to address barriers to CEnR currently posed by IRBs/HRPPs. While there is no one-size-fits-all solution to the types of barriers discussed here, the myriad of different strategies, at different levels and with different amounts of required resources, are likely to be useful to provide a sort of “menu” of types of approaches that could work at many institutions.

It also appears that many IRBs/HRPPs are in the midst of transforming to support CEnR. This is promising, yet real change takes time. We hope our findings motivate both IRBs/HRPPs and CE leaders to continue to transform, wherever they may be on that continuum of change.

It is important to note that barriers to effective CEnR, and strategies to address them, exist beyond the IRB/HRPP space. As noted above but not included in our analysis, some interviewees mentioned the importance of increasing both academic researchers’ and community researchers’ competency for CEnR. Beyond this, they also mentioned the importance of engaging with broader structural influences on CEnR, like funders, academic institution leaders, and department heads. The potential impact of these recommendations on academic-community research partnerships and institutional trustworthiness as well as on the quality of CEnR and direct benefits to communities merit future study.

There remains a lot of work to be done, and it will require ongoing collaboration between HRPPs and CEnR researchers. By highlighting common barriers and sharing strategies used by CE program leaders to address them, we hope these insights can serve to facilitate and advance the work of CEnR by advocates across different institutions. There are also important lessons to be learned from tribal IRBs and community-based research review mechanisms that can offer insights and alternatives to the traditional academic IRB model [21]. This can serve to meaningfully transform institutions to better support and advance the work of CEnR to advance translational science as well as community-identified research priorities.
